# Recovery of precious metals from waste streams

**DOI:** 10.1111/1751-7915.12759

**Published:** 2017-07-13

**Authors:** Jing He, Andreas Kappler

**Affiliations:** ^1^ Geomicrobiology Center for Applied Geosciences University of Tuebingen Tuebingen Germany

## Abstract

As there is a high potential for microbe‐based technologies to bring the recovery of metals from waste streams to an ecologically friendly and financially reasonable level, it is worth to invest efforts into the advancement of these biotechnologies in the future.

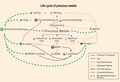

## Introduction

**Figure 1:**
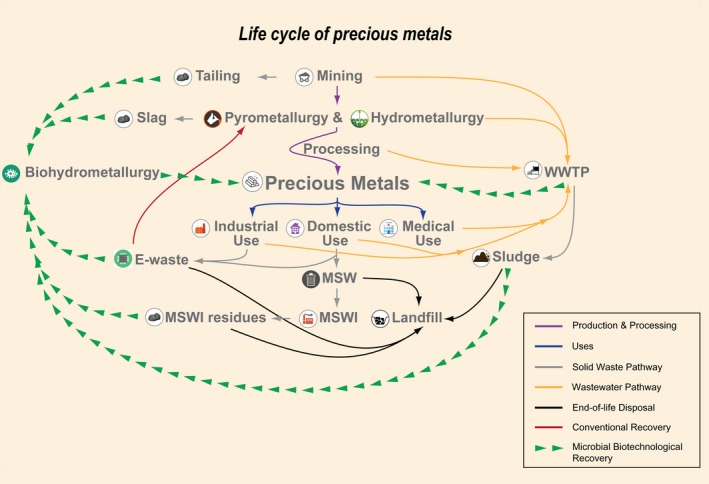
Life cycle of precious metals including the production and processing (purple), uses (blue), solid waste pathway (grey), wastewater pathway (orange), material end‐of‐life disposal (black), conventional recovery of precious metals (red) and possible microbial biotechnological recovery (green) pathways. (MSWI, municipal solid waste incineration; WWTP, wastewater treatment plant).

As the world population and global economies continue to grow at an increasingly fast pace, and due to advancements in science and technology, the demand for critical materials, including gold, platinum‐group metals and rare earth elements, is rapidly rising (Mack *et al*., 2007; Das, 2010; Morf *et al*., 2013). This demand comes at a price – the mining of such precious metals converges to generate risks to ecosystems and human health (Grandjean *et al*., 1999; Wong *et al*., 1999; Eisler, 2004). Conventional mining activities can result in the pollution of soils, groundwater and surface waters (Hutchinson and Whitby, 1974; Li *et al*., 2014). Such consequences are further compounded by the tremendous amounts of waste that are generated every day, predominately from construction and demolition, industrial and commercial activities and domestic households, leaving a legacy of non‐recycled metals in disposal facilities (Poon *et al*., 2001; Bambas‐Nolen *et al*., 2013). Microbial biotechnology offers a ‘natural’ way of addressing environmental issues ranging from bioremediation techniques to green biohydrometallurgical processes for industrial, agricultural and municipal effluents and residues. Microbial biotechnology is also a crucial element in the paradigm of ‘sustainable development’, which targets goals that include in particular a safe disposal of waste as well as the recovery of materials, for example precious metals, that are present in all kinds of waste streams from different sources (Fig. 1) (Moo‐Young *et al*., 1996; U.G. Assembly, Transforming our world, 2015). Using biotechnological approaches, precious metals can potentially be recovered from materials that are not economically beneficial by conventional techniques.

## Current obstacles to mining and recovery of precious metals from waste

Conventional processes of precious metal mining and recovery are costly due to the high consumption of energy and use of chemicals for metal mobilization, cementation, etc. To limit costs, and ensure profitability, industries therefore tend to use high‐grade ores as raw material (Marsden and House, 2006). However, mining activities are nowadays more and more constrained by limited high‐grade ores due to depletion of local resources coupled to restrictions in the import of precious metals as a result of laws and regulations of metal‐exporting countries (Fleming, 1992; Schubert *et al*., 2015). Therefore, recycling precious metals from waste streams is a possible solution that can alleviate the disparity between supply and demand.

Tailing dumps accumulated at gold mine sites, electronic wastes and other unconventional resources can be used to extract and recover precious metals (Natarajan *et al*., 2001; Gericke and Pinches, 2006; Reith *et al*., 2007). However, tailings containing gold‐entrapped sulfides with low levels of gold cannot be economically processed through conventional approaches. Gold particles that are locked up in host rocks make the recovery by gravity or cyanidation methods difficult or even unfeasible (Marsden and House, 2006). Although e‐wastes contain relatively high levels of precious metals allowing their profitable recovery, traditional pyrometallurgical and hydrometallurgical processes have several limitations. Pyrometallurgical processes (smelting) require high financial investments for energy and generate hazardous emissions (Cui and Zhang, 2008; Khaliq *et al*., 2014; Kaya, 2016). As such, cyanide leaching is a widely applied hydrometallurgical strategy to recover gold. However, a series of environmental accidents at various gold mines around the world has caused widespread concerns regarding the use of cyanide, which is highly toxic (Cui and Zhang, 2008).

Household and industrial wastes are currently mostly burned in incineration plants and subsequently disposed of in the environment or used as additives for construction purposes (e.g. in cement). This can be problematic due to potential leaching of toxic compounds into the environment and the loss of precious metals that remain in the waste. Over recent decades, there have been several attempts to recover valuable metals from such wastes by conventional methods including ageing, sieving, crushing, magnetic separation, density separation and eddy current separation (Chandler *et al*., 1997). However, these procedures do not effectively recover precious metals from such materials. The main constraint is the relatively low abundance of precious elements in the municipal solid waste (MSW) incineration residues and the high structural and compositional complexity of the incineration waste matrix (Mishra and Rhee, 2010; Morf *et al*., 2013).

Other waste streams also contain precious metals, such as wastewaters from plating processes. For example, sewers contain platinum (Pt) particles stemming from automotive catalysts, wastewaters from hospital (e.g. chemotherapeutic drugs containing Pt). The main limitation for recovering precious metals from those wastewaters is that the levels of precious metals are usually quite low (Bhagat *et al*., 2004; Ju *et al*., 2016). This means that conventional ion exchange resins, solvent extraction or reduction and precipitation methods of precious metal recovery by adding chemical reagents are not economically attractive. Therefore, there is a strong need to develop and apply low‐cost and eco‐friendly methods to recover precious metals. Strategies including microbially assisted recovery can potentially offer an affordable, sustainable approach to meet rising demands in precious metals (Fig. 1).

## Microbial biotechnological approaches to recover metals from waste

Biohydrometallurgy is based on the application of microbially catalysed processes for the extraction of metals from different raw materials. Biotechnological approaches cover all cutting‐edge areas of biohydrometallurgy, which include bioleaching, bioprecipitation, bioflotation, bioflocculation, biooxidation, biosorption, bioreduction, bioaccumulation and the application of biosensors in analytics (Sivasubramanian, 2016). At the early stage of applying bioprocesses at an industrial scale, bioleaching and biooxidation were widely used to extract metals from natural ores (e.g. sulfides) (Hunter, 2002; Watling, 2006). With the rise of environmental awareness in our society, people have started to expand the application of microbial biotechnology into waste treatment and metal extraction from waste. Such biotechnological approaches could not only reduce the toxicity of various waste streams, such as mining wastes, e‐wastes and even municipal waste sludge, but also unearth the potential of those wastes as secondary materials for precious metal recovery (Mishra *et al*., 2005; Shin *et al*., 2015; Awasthi *et al*., 2016; Palomo‐Briones *et al*., 2016).

In most cases, the metallic gold/silver recoveries achievable from tailings by conventional cyanidation methods are low. However, applying a pre‐treatment by biooxidation/bioleaching of gold‐ and silver‐bearing sulfide tailings before cyanidation markedly increased the extraction recovery of these precious metals from these tailings (Attia and El‐Zeky, 1989; Marsden and House, 2006). Locked‐up precious metals could be liberated by biooxidation of their sulfide matrix. Microorganisms including bacteria, fungi and yeasts can also recover soluble precious metals by biosorption and bioreduction (Das, 2010; Colica *et al*., 2012a; Maes *et al*., 2017). For instance, the fungi strain *Trichoderma harzianum* is been able to take up silver efficiently from metal‐contaminated waste‐rock dumps (Cecchi *et al*., 2017). Ruthenium contained in industrial effluents was shown to be recovered using purple non‐sulfur bacteria (*Rhodopseudomonas palustris* strains) as biosorbent (Colica *et al*., 2012b). The mechanisms of biosorption are generally based on physicochemical interactions between metal ions and the functional groups present on the cell surface, such as electrostatic interactions, ion exchange and metal ion chelation and complexation (Özer *et al*., 2005). Some microorganisms can reduce precious metal ions to their metallic forms. For example, sulfate‐reducing bacteria can reduce Pt(II) to Pt(0) (Riddin *et al*., 2009; Ito *et al*., 2016). A magnetotactic bacterium, *Stenotrophomonas* sp., can remove Au(III) from contaminated wastewater by reducing the Au(III) to Au(0) followed by deposition of nanocrystals of Au(0) particles on the cell surface (Song *et al*., 2008). Cyanidation processes in mining activities generate wastewaters containing complexed Au anions, i.e. Au(CN)_2_^‐^. Gold recovery from cyanide solution by a bacterial consortium was studied by Aitimbetov *et al*. (2005). The main mechanism underlying this process was the degradation of the metal cyanides by the microbial consortium associated with the release of gold ions. The Au was subsequently precipitated as Au(0) on iron and zinc reducing agents.

These microbial biotechnological approaches are promising for economically recycling of precious metals and have the potential to be not only cost‐effective but also environmentally friendly (Sivasubramanian, 2016). Recycling metals from wastes is an important subject not only from the point of the recovery aspect of valuable materials but also from the point of waste management. For example, it would be very beneficial if toxic metals can be easily removed from waste so that the waste can be used for secondary purposes, e.g. in construction.

## Likely contribution of microbial biotechnology to the recovery of precious metals from waste

Applications of biohydrometallurgy for extraction of precious metals from natural ore deposits have been developed and applied in a few cases (Chandraprabha *et al*., 2002; Sun *et al*., 2012). Yet, another application of microbial biotechnology is the bioprocessing of industrial and other wastes to first recover precious metals from the waste and second to add value to a ‘clean’ or at least a less toxic waste product (Cui and Zhang, 2008; Ilyas *et al*., 2010; Maes *et al*., 2016). The availability of high‐grade ores of precious metals on Earth is not endless, and the ongoing consumption of these limited resources will eventually lead to a rapid depletion of high‐quality ores. By viewing metal‐containing waste as secondary ‘ores’, we can avoid exhausting natural resources. Industries can potentially also enhance their profits by applying microbial biotechnology processes, which can be cost‐effective and have a low energy requirement. In addition, several environmental benefits could be achieved. First, treatment of waste by such bioprocesses can reduce the total amount of waste. As a consequence, land space for waste storage and disposal could be saved for other human activities. Second, microorganisms can be applied to destroy or degrade toxic compounds (e.g. cyanide) (Akcil and Mudder, 2003), or perform bioprocesses that are more environmentally friendly as no or less toxic chemicals are employed (Zinke and Gabor, 2009). Third, bioprocessing can efficiently recover valuable metals concomitantly to detoxifying the processed wastes (Natarajan, 2013). Finally, some solid waste materials (e.g. MSW incineration solids, coal fly ash) can be used as secondary materials for construction purposes, in particular once their toxicity is reduced through microbial bioprocessing (Hedrich *et al*., 2015). These waste materials could be used in a more sustainable way without creating severe environmental risks.

However, in reality, there are several obstacles in applying microbial biotechnology strategies to recover precious metals from waste. First of all, waste materials generally have heterogeneous textures and compositions. Additionally, the waste is very different in different regions of a country and varies from day to day, or from winter to summer months depending on seasonal behaviour of the population. Therefore, it remains challenging to find suitable microorganisms that deal with this heterogeneity and varying composition and texture effectively. A potential approach to this problem is to use microbial communities with many different microorganisms of varying functional capacities able to adapt to varying geochemical and mineralogical conditions of the waste material. Additionally, in the future, new strains or microbial consortia have to be isolated that are resistant to higher metal concentrations; this also includes the application of genetically modified microorganisms. Second, the utilization of microbial biotechnology should be adapted to the respective social, economical and technical requirements and conditions of certain areas. For example, if the local situation does not allow land space for bioleaching, microbial biotechnology approaches that are based on space‐requiring heap leaching methods are not suitable. Finally, if the recovery of metals using biotechnological approaches cannot be improved significantly (regarding the yield and/or purity of extracted metals) compared to more common physical and chemical methods, the financial investments needed are too high to make the biotechnological approach economically feasible. In summary, these challenges described suggest that there are still many hurdles to overcome to implement and apply biotechnological approaches on a large scale for recovery of precious metals from waste. Nevertheless, as there is a high potential for microbe‐based technologies to bring the recovery of metals from waste streams to an ecologically friendly and financially reasonable level, it is worth to invest efforts into the advancement of these biotechnologies in the future.

## Conflict of interest

None declared.
